# The Endoscope, and Its Application to the Diagnosis and Treatment of Urinary Affections

**Published:** 1867-07

**Authors:** A. J. Desormeaux


(*Continued from page 288.*)

The disease may long remain in this condition; the granular ulceration may exist at the same time that there is decided stricture, as I have told you, and as you have opportunities of seeing for yourselves. So long as the endoscope shows the characteristic ulceration, we are apt to hope, in causing it to disappear, that the disease is cured. But it sometimes happens, that the consecutive lesions, already well rooted, continue after the cause has disappeared, and strictures, often, are only curable by dilatation. Thus, the affection consists in two elements: stricture, to be evercome by mechanical means; and ulceration, perceivable by means of the endoscope, which, if cured by proper means, will soon bring on, or reproduce, the retraction.

This may explain one fact, often observed, and of which I vainly sought an explanation, until the endoscope enabled me to follow the disease in all its phases.

I have seen recent strictures, not extensive, easily dilated. After a few days they disappeared, but, the treatment stopped, they soon reappeared, and as often as they were dilated, so often did they recur, upon the cessation of the dilatation. Uselessly, have I continued this treatment; I have kept a healthy calibre for months, but, once the dilatation stopped, the stricture would return. I have concluded that new strictures are more apt to recur than those of more ancient date; the cause of this I cannot explain. I held this opinion, in common with many others, equally ignorant as to the cause. At present, the explanation is easy, as you have already seen. The ulceration, which continues, reproduces the swelling. Its persistence, formed upon a plate of chronic eczema, may, by dilation, be soon removed, to come back, so long as the eczema is not cured.

When the thickened parts disappear simultaneously with the discharge, the cure is complete. You recollect the case of D., cured by a single cauterization, and remaining cured for eight years. Many other cases, similar, are present in my memory, and you will see them at each consultation. For a long time, the stricture was not attacked directly; the only treatment was applied to the granulations. When the alteration of the tissues continues so long as to persist independently of the producing cause, such is not the case, and dilatation should be added to other treatment, In such cases, relapse is frequent; exceptions to this rule are rare, I know of but few. If the patients did not, as a general rule, disappear after treatment, such cases would be less rare.

We now reach the third period of the disease: the granulations disappear, as do those of the conjunctiva, when they have destroyed the structure of that membrane. Possibly, the obliteration of the vessels may play a great part in the case. But as the granular affection passes off, the stricture keeps up its onward march upon the retraction of the fibrous tissues extending to the mucous, the mechanism of which M. Guérin has well shown, the mucous and sub-mucous tissues take on a character cicatricial in its results. Before informing you as to the results of the endoscopic examination, I will relate to you the details of an autopsy which enabled me to study the disposition of this inodular tissue. The stricture never having been treated, the alterations found were evidently the result of the affection itself.

The patient entered my wards, in the Necker Hospital, with an affection of the foot, necessitating the amputation of a toe. During his sickness he told me that he had had a blennorrhagia which had never been treated, and the result of which was difficulty in emitting the urine. After curing the foot I found a stricture.

The patient died of purulent infection, and the autopsy showed metastastic abscess in the lungs, and also the following lesions in the urethra:—

The portion of the canal strictured is generally the bulbous, and the extent of the stricture is about half an inch in length (2 centimètres). In this portion, the color is of a whitish or slightly yellowish color, whilst the remainder of the urethra is very red, rough, and of the appearance generally seen in corpses. The diseased mucous is attached to the subjacent membrane, from which it is not easily detached, the layers of which are thicker than natural. Below the cavernous part, the bulb presents a healthy and natural appearance. Microscopically examined, the mucous membrane shows an epithelial layer, formed by cells more irregular than the rest of the urethra. Below this epithelium, the mucous tissue affords fibres similar to those in a healthy state, but mixed with abundant elastic fibres, many cytoblasts, and granulations such as we find in the primary organization. The sub-mucous tissue is especially formed of bundles of fibres, in the intervals of which the cytoblasts and granulations are very abundant, many more than in the mucous; there are also many elastic fibres and fibro-cellular cells in different stages of granulation.

This confirms the general opinion as to the inodular character of stricture of long standing, where caustic had not been used; no traumatic cause was present, and yet the elements of cicatrization and the fibro-elastic element were both more abundant than in the normal state.

The identity existing between this formation and that of the cytoplastic tissue, readily explains how strictures of long standing, and organized, may rebel at dilatation, be retractile, and present the same appearance of elasticity as do cicatrices. Really, the structure is the same, and, paradoxical as it may seem, the stricture is, at its last period, less a disease than the effects of anterior treatment for a disease; so the inodular filaments, no matter the trouble caused, are only the result of a wound.

Similarly endowed inodular strictures resist dilatation, only obtainable by much time and much fatigue to the patient. This is not, however, the greatest inconvenience. The urethra once brought back to its original size, once the treatment stopped, the retraction reappears and the stricture returns. In severe cases, a few days may produce that which weeks or months will not replace. There is no reason to hope, in such cases, for a durable cure, by prolonging the use of dilating instruments, for in such cases, as in the sequences of burns, the cicatrices object to extension. In extreme cases, the patients may not be cured, but a bougie may be passed now and then. They must be placed upon a continuous treatment, not to be interrupted without being forced to recommence the dilatation. A similar case is scarcely bearable, in addition, once the malady has taken its course, urethral inflammation, or vesical, urethral fever, or other accidents will aggravate it and render it more difficult to treat.

These are the reasons causing us to admit the principle of incision in the third degree of stricture. It is well known that the resulting cicatrix is thinner than cut tissue, it does not reproduce the stricture, and enables the patient to introduce the bougie at long intervals.

From what we have said, it results that there are three different kinds of stricture, to be met by different treatments. In the acute inflammatory, cure the blennorrhagia; in the chronic inflammatory, it must be, for a long time, against granulation, but later, dilatation comes into play; in the inodular, it is necessary to attack the fibroid tissue and, consequently, fall upon an appropriate treatment.

These indications admitted, the endoscope enables us to see, at once, the character of the stricture.

### FOURTH LECTURE.

### *Inodular Stricture.—Urethral Fistulas.*

Gentlemen:—Before touching upon the treatment of inodular stricture, it is necessary to acquaint you with the means of exploration which can furnish the indications for the therapeutics demanded in their treatment.

The means of exploration usually employed, furnish a certain number of useful signs, both for diagnosis and for treatment. We must expect the same from the endoscope; it does not double other means, as I have already told you, it only adds to their efficiency, in enabling us to complete the study of the disease. Other instruments furnish all the knowledge that is attainable by mediate touch (*toucher*), the endoscope adds to them that knowledge derivable from sight.

The sound, ordinarily, reveals the existence of a stricture and its seat; even if it passes one stricture, it can tell us if there are others beyond it. Simple bougies, bougies with bullet ends, and other similar and analogous instruments, may acquaint us with the degree of retraction of the strictured point. The ball-pointed instruments enable us, moreover, to determine the number and extent of the strictures.

The bougie and the curved stylet will also, sometimes, enable us to judge upon which side of the coarctation the opening may be found, this by the direction it may be necessary to give the instrument for the purpose of meeting it. Also, the consistency of the fibroid tissue may be appreciated by the more or less difficulty experienced in passing a conical bougie, the point of which has entered into the channel still left in the stricture; its elasticity, by the resistance experienced on endeavoring to reintroduce it, after having once withdrawn it; its retractility, by the rapidity with which it loses its acquired diameter, when dilatation has been suspended.

Such are about the opinions drawn from the means generally employed. It is, I think, useless to add to them the results of explorations made with bougies for impression (à empreintes). It is useless to repeat here, all that has been said of the fallacies they are guilty of; the little use made of them at present is sufficient evidence of their uselessness.

To resume, the different means employed by the surgery of the present time enable us to appreciate:—1st, the existence and seat of the stricture; 2d, their number; 3d, their extent; 4th, their calibre; 5th, their elasticity; 6th, their retractility; 7th, their consistency.

To these, the endoscope enables us to add coloration and the configuration of the surface anterior to the coarctation, and, also, the exact position of the orifice. It also affords us the means to decide at once if it is inodular, and also to positively say what should be the treatment.

We have seen that at the intermediary stage, the mucous membrane loses its red color; and also its ulcerous appearance, at the same time the stricture is being organized; it can also retake its normal appearance.

This condition, which has terminated with the second period, changes at the commencement of the third. With length of time, the infiltrated lymph continues to organize; the tissues increase their inodular appearance, and, when examined by the endoscope, instead of the rosy color of the healthy urethral mucous, or of the red color presented at the commencement of this stage, when irritated by the introduction of instruments, the retracted point shows a paler hue than natural, and one which becomes yellowish or greyish, often dull, sometimes mother of pearl (*nacre*), and simulating the cicatrices that are the sequences of burns, and such as the pathological anatomy of stricture enables us to describe.

The configurations presented at the end of the endoscope by inodular stricture, vary much, as do all other pathological cases in the case of disparritees, still we can form them into almost three principal classes.

Some commence funnel-shaped, in which the most retracted point is at the lower extremity of the coarctation. Unfortunately, this is rare in hard or in ancient strictures, because it facilitates the introduction of the instrument. Another form, already recognized in the preceding stage, and often occurring in this, is the mamellonated. In this we find, at the end of the sound, mamelons formed of indurated tissue, readily discovered by the resistance offered to the introduction of the instrument, with difficulty bent upon their surface. It may happen that the mamelon is unique, circumscribed by a groove and tongue, causing the introduction of the sound to be difficult, the opening being hard of access, generally found in the middle, and opposite the salient point of the mamelon, but sometimes to its side. Generally, the orifice is where it appears most large and most deep.

If there are several mamelons, their summits verge towards the centre, and it is towards their convergence that the opening is found. Sometimes, these mamelons are so irregularly disposed that the orifice is hidden from all researches made with the stylet. In such, the bougie is often difficult to introduce, because it does not find the funnel-shaped orifice which will direct it.

Sometimes, between the affected points, there are parts more or less healthy and vascular, showing more or less red or inflamed tissue.

Finally, I may say, that in a last form of this disease most rebellious to catheterism, by the ordinary modes of treatment, the sound showed an obstacle like a diaphragm, the surface of which showed plates unequal, anfractuese, with many small anfractuosities and some greater ones; the eye could not discern all, and especially those which corresponded to the entrance through the stricture. To recognize it, we must examine these depressions mechanically, until we find one softened. But when the stricture is hard, the stylet does not free it readily, even after several efforts, though it may have entered the retracted canal.

There is a variety of this form which I have rarely seen, and always in old and hard strictures. In this, the orifice, be it situated in the centre or other parts found at the point of the sound, is gaping, and simulates a small rounded opening, into which one could easily introduce the end of the stylet. But, though the stylet or the whalebone bougie might be readily introduced, it is often very difficult to pass through the contraction, and especially to dilate it; in those cases the tissue is very resistant.

In addition to the color and configuration of the obstacle, the sound pushes them before it, instead of repressing, as in cases of healthy or simply inflamed mucous membrane. This sign is especially useful in those cases where the transformation, commencing in the exterior tissues, has not yet reached the mucous surface, still red or ulcerated, and thus we may think the evil less advanced than it really is. But, in such case, touch, by aid of the sound, exposes to us, under the red surface, an indurated and resisting base; it is thus evident that the inodular tissue has formed, though not yet come to the surface.

It has been stated that sudden strictures are caused traumatically, and are thus distinguished from those caused by blennorrhagia, of which the anterior portion is funnel-shaped; but the endoscope shows us that these last often come on suddenly, and the first may be gradual.

Whatever may be the appearance of the coarctation, it does not change on the withdrawal of the sound, because the tissue has not lost its suppleness and cannot pleat and close over the end of the instrument. On withdrawing the canula, the healthy parts close over its end, like a curtain, but the indurated parts remain unchanged in appearance.

You see, gentlemen, the facility and precision the endoscope brings to the diagnosis of inodular stricture. Without this instrument, we could only admit them, except in traumatic cases, by reason of the continuance of the malady, or of the difficulty of dilatation and the rapidity of the return of the stricture, but the duration furnishes only deceptive probabilities, for if strictures caused by blennorrhagia are sometimes fibrous after a few months, you may remember that in the case of D., spoken of in a former lecture, he, after a continuance of the affection for eleven years, had only a chronic inflammatory coarctation, which disappeared with the granulations; and, in another case which I have reported, the disease yielded rapidly to dilatation and a treatment of the ulceration still existing. Then there only remains, as a sign, unsuccessful dilatation, and so we can only after unsuccessful treatment and lost time recognize the lesion. By aid of the endoscope, on the contrary, we can immediately tell the differences; whenever there is found at the end of the instrument, an inflamed or ulcerated instead of a healthy membrane, a white surface, mamellonated, anfractuous, or of fibroid appearance, it is certain an inodular stricture is present, and the therapeutical indications are immediately understood.

We will now take up the treatment of the coarctations, and of the indications to be met. They consist in two principal ones:—1st. Pass through the stricture; 2d. Destroy the obstacle.

*1st.—Traverse the Stricture.*

This is often easily done; it is only necessary to introduce a sound or bougie, sufficiently fine to pass through the obstacle. If the opening is not readily found, the bougie is to be drawn back and again gently readvanced, with these precautions it generally occurs that the point of the instrument will, at some moment, gain the opening, then, gently pressing it onward, it reaches the bladder. I shall delay no longer on this point, which is not exactly allied to the subject at present under consideration. Success almost always attends it, but there are cases where patience and hability, no matter how great, fail to discover the opening, the point of the bougie will butt against the surface precedingly described, and, do what we may, it will always stop there. After continued efforts, the attempt is deferred to another day, when the insuccess is the same; even days and weeks may pass in futile attempts and in failure. Many means have been recommended for such cases: whalebone bougies, bougies very fine, introduced side by side, so as to cover the whole obstacle with points, one of which might be lucky enough to pass, the sound open at both ends (à deux bou) passed up to the retraction, to serve as a conductor of a fine bougie. I have tried all of these means, they sometimes succeeded, but I prefer a conical bougie, twisted at its point; but I must add that, in some of these cases, none of these treatments are successful. It is doubtless rare that, with time and patience, success does not follow; but patience has its bounds, and we will soon come to cases where it has been necessary to relinquish futile attempts at cure.

These fruitless attempts cannot be long continued without danger, and especially so when the urine does not flow at all, or only by driblets; the distended bladder threatens rupture, the patient experiences intense anxiety, the urinary infection manifests itself; or if less severe, the distension less prolonged, it may at least cause inertia or paralysis of the organ again, the superior portion of the urethra becomes fatigued, inflames, softens, urinary abscesses form, which, after many dangers, result in urinary fistulas. In such cases, not very common, temporization and continuous treatment will not answer. It is necessary to act, and to act quickly. What shall we do? Surgery has but one resource, the puncture of the bladder, because forced catheterism is properly rejected for all cases of this kind on account of the bad effects it causes. The puncture of the bladder is made but, say what you may, experience teaches that it is not without danger. Its effects are, moreover, only temporary, they do not reach the cause, which still continues, and if the coarctation remains, the danger is from one day to another. This was the case with the patient whose history I will give you:

For several years he had a stricture, which followed the usual march, increased more or less rapidly, and finally caused complete stoppage of the urine. The patient placed himself in the hospital, under the care of one of the most able of our masters. At the time of entrance, the bladder reached as high up as the umbilicus, the danger was great, the fever violent, and pain and tumefaction had already reached the perineum.

All attempts at catheterism were fruitless, and the incision of the swollen perineum bringing off but little water, hypogastric puncture of the bladder was resorted to. This produced ease, but the stricture was not relieved; it so happened, however, that the urine passed, slightly by the urethra, and much by the punctured fistula, the puncture in the hypogastrium having healed, the patient went about his business. Soon, however, the urine ceased to pass through the canal, and the fistula becoming more contracted, the bladder could not empty itself, and so the patient entered Cochin Hospital, of which I was then the surgeon. This was in 1861, when I was experimenting with the endoscope, but had never yet used it for the purpose of passing strictures. The fistula, though not permitting sufficient urine to pass, was still sufficient to postpone danger, and for several days I tried to introduce the smallest conical. bougies. In this I was neither more expert nor more fortunate than my former teacher. I then decided to try the endoscope; by its aid, I perceived the obstacle and an opening into which a stylet could be passed, then I had not thought of the whalebone bougie (this patient caused me to imagine it). I withdrew the instrument, and, after several efforts, a fine bougie (filiform) traversed the stricture, previously passed by a sound. This was much, but for a long time I was compelled to employ the endoscope as a preparation for the bougie. At length the dilatation went on regularly; but when bougies of a low number readily passed, the patient pressed by his vocation, and satisfied with his then condition, wished to leave the hospital.

The following year, 1862, he found me in the Necker Hospital. The same fistula existed, and the incompletely dilated stricture had returned. I had uselessly wasted time in catheterism without the endoscopic aid, but now I concluded to combine the two. I employed endoscopic urethrotomy, and the obstacle was removed in less time than we had previously spent in vain attempts. We will return to the subject of urethrotomy, but I will state that in this case, in absence of the endoscope, I would have been compelled to puncture the bladder, as had been done before, or else to an incision (boutonnière), that is to say, to urethrotomy without a conductor, the difficulties and dangers of which I will not now recall.

This case will enable you to appreciate the utility of the endoscope as a means of passing difficult strictures. Now for the manner of its use as I employ it:—

The endoscopic sound is passed forward to the obstacle, and, the instrument being arranged, we can perceive the condition that I have described, and, frequently, the orifice in the retracted part. In this examination, care must be taken to keep the sound in the direction leading to the affected part, so that its anterior face coincides, through its whole extent, with the end of the instrument. As the examination is almost impossible if the instrument is vertical, it is necessary to place the patient in such position that the axis of the diseased part nearly assumes the horizontal line. To act upon the membranous portion, dorsal decubitus is preferable; but for the bulb, and especially its anterior part, it is best to raise the patient to almost a sitting posture. When the diseased surface is thus brought fully before the end of the sound, the orifice is almost always in the field of the instrument, but it sometimes hides itself under the edges of the wounds; then the direction must be changed to discover it. Frequently, in like cases, I have found it towards the lower portion of the canal, and the indurations existing upon the superior portion.

The orifice once found, a stylet must be introduced, to make sure that we are right, and also for the purpose of traversing the retracted part. This is easier done with a metallic instrument than with the whalebone bougie, which is not so conveniently managed. The stylet usually employed is shaped like the ordinary probe, mounted upon an elbow-shaped handle (manch coudé) of the forceps character (en form de palette), by which it is held at the exterior of the sound; it is well to have at least two of different sizes.

The patient being then placed in the proper position, and the penis held by an assistant, who at the same time may steady the sound in the axis of the canal and firmly hold the parts, the stylet is then introduced through the lateral opening in the sound, and we try to make it enter into the orifice of the coarctation. I have already said that, if the snrface of the obstacle is irregular, with several depressions, try each until one is found that the probe will enter. Sometimes, the first attempt is successful, and the instrument is only arrested on the farther side by a mucous pleat; but sometimes it stops after a short entrance, in the sinuosities of the stricture, which rarely furnishes a uniform calibre. In such cases, the stylet may be carried in different directions, and we may even move the sound so as to permit the passage of the probe. Once the obstacle passed, it can be dilated by changing the stylet for a bougie, which can remain *in situ*. We can, as I have just said, withdraw the endoscope, and try to introduce an elastic bougie; this is, however, an uncertain process, and it is best, whilst the endoscope is in place, to take advantage of it for the purpose of well placing the bougie.

For this, a whalebone bougie must be used, soft ones cannot be properly directed. I have sometimes used bougies of catgut, but they are worse than whalebone. These should be elbowed like the probe, so that they may be kept at the sides of the sound. We then substitute the whalebone bougie which we cause to follow the same way. Like the first, it sometimes stops after passing the stricture, because it catches in the curvature of the urethra. Without effort to go farther, the endoscope is to be withdrawn, leaving the probe in its place. To do this, press it gently whilst the instrument is retracted. Once isolated, it can be made to enter the bladder; but, in difficult cases, where success is not immediate, fix it in its position, and, after a few hours, it rarely happens that the attempt does not succeed. I will not rest on the subject of dilatation, only saying, the first bougie must give way to a larger, patience and dexterity are alone necessary. To demonstrate to you the advantages of the endoscope, and especially where ordinary means fail, I will recount a case of M. Civiale, with his given permission;—

*Traumatic Stricture.—Futile Attempts at Catheterism.—The Introduction of a Bougie by means of the Endoscope.*

M., (Paul,) aged 36, a cook, and a patient in the Necker Hospital. The patient is healthy, has never been very sick. When about 28, he contracted a blennorrhagia, which was well cared for and cured.

About five years since, he slipped on the stairway and wounded the perineum; a physician, called at the time of the accident to stop the abundant bleeding, tried, fruitlessly, to pass a catheter; an able surgeon, later called upon, was not more successful. The hemorrhage ceased of itself, the patient easily urinated, the jet was notably diminished, but the patient did not complain.

Some months afterwards, the diminution of the jet became great, and soon the urine was discharged only by drops; later on the discharge was involuntary, and this condition continued for about four years. As a consequence of excessive work, total retention came on, and it lasted two days.

The patient then decided to enter the hospital in which the surgeon who had ineffectually attempted to sound him was on service. A stricture of four months standing, under the pubic arch is found; it cannot be traversed. Urethrotomy is practiced; the operation lasts twenty minutes; the opening into the canal cannot be found; the wound is soon cicatrized.

Shortly after his exit from the hospital, as a consequence of drink, it returned, with fever, retention, loss of mind, and delirium. Catheterism was again vainly essayed.

Three days after, Dec. 22d, 1862, the patient came to the Necker Hospital, with slight fever, sleepiness, and small appetite; the urine passed only by drops, and when he was up the passage was involuntary. M. Civiale tried for twenty-eight days, without success, to produce a cure. Bougies of all kinds and of all sizes, wax, whalebone, caoutchouc, and metallic, all in vain. These bougies and sounds could never strike the opening. No accident occurred and not much pain was felt.

*8th Jan.* I, with the help of the endoscope, tried to find the opening in the stricture; a medium-sized canula was readily introduced to the retracted point; the membrane was white and shining, cushion-shaped (*bourrelet*); many depressions were also seen, which I tried to pass with the probe and with bougies of whalebone; the attempt was futile, the pain great, and further examination postponed.

On the *11th Jan.*, I found an orifice, into which I introduced the stylet, to the extent of about one-third of an inch; the whalebone bougie was left *in situ*. On the morrow there were some symptoms of fever, which disappeared the next day; there were also vague pains in the region of the right kidney.

On the *13th Jan.*, the stylet was again introduced and penetrated farther; a whalebone bougie was left in its place; in the evening it became deranged, and the nurse, in attempting to bring it right, passed it into the bladder, without difficulty or pain.

The bougie was fixed and kept so until the *19th of January*, when, during the night, slight fever and difficult urination caused the patient to withdraw the bougie, which was the next day replaced by a small sound. The patient experienced no inconvenience from the presence of the sound; the urine passed between the sound and the canal.

On the *29th Jan.*, the sound was withdrawn and a larger one inserted.

All efforts were made by those whose ability none could doubt, and unsuccessfully. Then, M. Civiale asked me to try the endoscope, thinking, with me, that if it failed, there only remained urethrotomy to be resorted to, and you all know the result of that operation. I found the orifice, and proposed to to incise upon the stricture, but M. Civiale preferred the means he generally employed.

I will no longer press upon you attention the advantage of the endoscope in such cases; you must have seized them before now. But there are other cases, where this instrument has enabled me to avoid opening the bladder either by puncture or by the knife.

The obstacle once overcome, dilatation by bougies may well be employed, and I have long used this method. At first sight it seems the least dangerous treatment, if used gently and patiently, but a more careful investigation will cause another opinion. In inodular strictures, the dilatation often requires time; at the commencement, especially, it may be necessary to pass the same bougie several times without changing it for a larger one. Moreover, the treatment may bring on an attack of fever; some patients are subject to it after the most easy catheterism. The sulphate of quinine, given at each introduction, will, necessarily prevent this. But if this course has to be persevered in, the treatment will not do. In other cases, urethritis, cystitis, and nephritis are a consequence; and when resumed after several days, we find we have lost part of what we had gained, the tissue has again retracted, and we are lucky if we do not find the parts in the same condition in which we commenced the treatment. Well, suppose these accidents do not occur, or, after having passed them, is the patient nearer a cure? No! Many of our patients have been cured several times. Really, these retractions are, as I have told you, inodular in form and analogous to a cicatrix, and, when once dilated, they return as do the contractions occurring after a burn. The following observation will show this:—

*Coarctation of the Bulbo-Membranous Portion of the Urethra.—Dilatation.—Rapid Relapse.—Urethrotomy.—Cure.*

B., (Louis Joseph,) aged 60, potter by trade, was in the

Necker Hospital. He was pale, thin, with soft flesh; had been a soldier in Africa; had had tertian fever; also, at 36, clap, treated and cured, at the end of six weeks, by injections of sulphate of zinc. No new case was contracted, according to his account, since then. From time to time, he did drink too much, but otherwise his life was regular. Some five or six years since, he was retaken with his tertian, at Paris, and entered this hospital. He was treated with the shower bath.

About twelve or fifteen months ago, he first observed a difficulty in his micturation; he could not long retain his urine, and, whenever pressed, was forced to evacuate it at once. Gradually the jet became finer, of less force, and, notwithstanding his efforts, it was difficult to completely empty the bladder, even after long, continuous attempts. These symptoms were most marked after his meals and especially so if he had taken too much wine. He, fearing catheterism, never was willing to subject himself to treatment.

On the morning of the 29th November, upon waking up, he could not pass one drop of urine, and could not attribute this misfortune to any cause. He remained in bed, with slight and repeated chills, nausea, great thirst, pain in the gland, the perineum, and great tenderness in the belly. Thus he passed the day at home, hoping that he would soon be well, but the symptoms increased in violence and about midnight he concluded to ask hospital aid. The assistant on duty tried to introduce a metallic sound, which was stopped in the perineal region. A considerable hemorrhage occurred, the patient returned to his home, and the next morning came to the consultation.

*30th November.* The patient was much frightened and suffering, there was also great pain caused by the pressing desire to make water; the least movement caused a revival of the intense pains, his face was contracted and anxious, his mouth dry, his belly tender and swollen from the effects of the distended bladder, which reached the umbilicus. The testicles were retracted towards the inguinal ring, in half erection, slightly turned upon themselves; the pulse was accelerated; the respiration irregular and rude. I introduced a conical bougie No. 4, which, after

